# Effects of Aging on the Neural Mechanisms Underlying the Recollection of Memories Encoded by Social Interactions With Persons in the Same and Different Age Groups

**DOI:** 10.3389/fnbeh.2021.743064

**Published:** 2021-09-10

**Authors:** Eri Tsuruha, Takashi Tsukiura

**Affiliations:** ^1^Department of Cognitive and Behavioral Sciences, Graduate School of Human and Environmental Studies, Kyoto University, Kyoto, Japan; ^2^Graduate School of Advanced Integrated Studies in Human Survivability, Kyoto University, Kyoto, Japan

**Keywords:** fMRI, source memory, aging, generation, social interaction, hippocampus, anterior temporal lobe, superior temporal sulcus

## Abstract

Memories related to ingroup members are remembered more accurately than those related to outgroup members. However, little is known about the age-dependent differences in neural mechanisms underlying the retrieval of memories shared with ingroup or outgroup members that are categorized by age-group membership. The present functional magnetic resonance imaging (fMRI) study investigated this issue. Healthy young and older adults participated in a 2-day experiment. On the first day outside fMRI, participants were presented with words by unfamiliar persons in movie clips and exchanged each word with persons belonging to the same age group (SAG) or different age group (DAG). On the second day during fMRI, participants were randomly presented with learned and new words one by one, and they judged whether each word had been encoded with either SAG or DAG members or neither. fMRI results demonstrated that an age-dependent decrease in successful retrieval activation of memories presented by DAG was identified in the anterior temporal lobe (ATL) and hippocampus, whereas with memories presented by SAG, an age-dependent decrease in activation was not found in any regions. In addition, an age-dependent decrease in functional connectivity was significant between the hippocampus/ATL and posterior superior temporal sulcus (pSTS) during the successful retrieval of memories encoded with the DAG people. The “other”-related mechanisms including the hippocampus, ATL, and pSTS with memories learned with the outgroup members could decrease in older adults, whereas with memories learned with the ingroup members, the “self”-related mechanisms could be relatively preserved in older adults.

## Introduction

Memories for persons who belong to the same age group (SAG) as ingroup members are remembered more accurately than memories for persons who belong to the different age group (DAG) as outgroup members (for review, see Rhodes and Anastasi, 2012). This enhancing effect on memory is known as the own-age bias, which is a type of intergroup bias (for review, see Molenberghs and Louis, 2018). Previous studies have consistently demonstrated that faces, words, and behaviors related to ingroup members are perceived and remembered more accurately than those related to outgroup members (for review, see Molenberghs and Louis, 2018). In addition, there is functional neuroimaging evidence that recollection-related processes, such as association memory or source memory, are significantly disturbed by aging, and age-dependent disturbances in recollection are caused by decreased activation in the hippocampus (for review, see Shing et al., 2010; Koen and Yonelinas, 2014). However, little is known about how neural mechanisms underlying the recollection of memories related to the SAG people as an ingroup member and the DAG people as an outgroup member are changed by aging. To address this issue, the present functional magnetic resonance imaging (fMRI) study investigated age-related differences in task-related activation and functional connectivity during the recollection of memories encoded by social interactions with persons belonging to the SAG people as an ingroup member and the DAG people as an outgroup member.

Neural mechanisms during the processing of information related to ingroup members are shared with neural mechanisms during the processing of information related to “self” (for review, see Northoff et al., 2006), whereas neural mechanisms are common between the processing of information related to outgroup members and of “other”-related information (Golby et al., 2001; Rilling et al., 2008; Greven and Ramsey, 2017). For example, people belonging to the ingroup are regarded as the social self, which involves the dorsomedial prefrontal cortex (dmPFC; Volz et al., 2009) or precuneus (Scheepers et al., 2013). In addition, functional neuroimaging studies have shown that functional connectivity between the memory-related hippocampus and the self-related cortical midline structures (CMS), which include the dmPFC and precuneus, contributes to the self-reference effect known as the memory enhancement of self-related information (for review, see Murray et al., 2015). The importance of hippocampus-CMS interactions in the self-reference effect on memory was also reported in a social context, in which memories for unfamiliar faces encoded with the anticipation of future friendship with self were significantly enhanced compared to face memories encoded with the anticipation of friendship with others (Yamawaki et al., 2017). Regarding the processing of information related to outgroup members, one fMRI study demonstrated that the posterior superior temporal sulcus (pSTS), reflecting social cognition of others (for review, see Patel et al., 2019), showed significant activation in prosocial behavior toward outgroup members compared to those toward ingroup members (Do and Telzer, 2019). In addition, activation in the right hippocampus and the right pSTS, which reflects “theory of mind” related to the process of inferring mental states in others, was significantly identified in the competition with other persons compared to that with machines (Polosan et al., 2011). Significant activation in the right pSTS has been found when perceiving social information such as faces (Dasgupta et al., 2017) or when watching movies of faces with rich social information (Pitcher et al., 2011). Thus, memories of ingroup members could involve interacting mechanisms between the self-related CMS and the memory-related hippocampus, whereas interactions between the right pSTS related to social cognition for others and the memory-related hippocampus could contribute to memories of outgroup members.

Episodic memory is disrupted by aging (for review, see Tromp et al., 2015; Nyberg, 2017), and age-dependent decreases in the recollection of episodic details have been linked with disrupted function of the hippocampus in older adults (for review, see Shing et al., 2010; Koen and Yonelinas, 2014). For example, one fMRI study reported that recollection-related activation in the hippocampus age-dependently decreased, whereas familiarity-related activation in the entorhinal and perirhinal cortex was relatively preserved in older adults (Daselaar et al., 2006). An age-dependent decrease in hippocampal activation was also identified in the recollection of autobiographical memory (St. Jacques et al., 2012). In addition, there is functional neuroimaging evidence that an age-dependent decrease in activation and functional connectivity related to memories for face-name associations has been found in the anterior temporal lobe (ATL) as well as the hippocampus. For example, an age-related decrease in correlations between hippocampal and ATL activation was observed in the retrieval of social knowledge (name or job title) associated with faces (Tsukiura et al., 2011). Another study using transcranial direct current stimulation (tDCS) demonstrated that the retrieval of face-name associations was significantly improved by tDCS stimulation over the left ATL in both young and older adults, whereas tDCS stimulation over the right ATL had a beneficial effect on the retrieval of face-name associations only in young adults (Ross et al., 2011). In neuropsychological studies of brain-damaged patients, patients with ATL atrophy were significantly impaired in perceiving faces or voices of familiar persons (for review, see Gainotti, 2013; Cosseddu et al., 2018). Taken together with previous findings, an age-related decrease in the functional network between the self-related CMS and the memory-related hippocampus-ATL regions could be critical in age-related decreases in memory for faces belonging to the SAG people as an ingroup member, whereas the aging effects on memory for faces belonging to the DAG people as an outgroup member could be modulated by age-related decreases in the functional network between the other-related pSTS and the memory-related hippocampus-ATL regions. However, little is known about the neural mechanisms underlying age-related differences in the retrieval of memories shared with ingroup and outgroup members that are categorized by age-group memberships.

To address this issue, using the event-related fMRI technique, we scanned healthy young and older adults during the retrieval of memories shared with the SAG people as ingroup members and with the DAG people as outgroup members. On the basis of previous studies, we made two predictions. First, activation in the dmPFC, precuneus, hippocampus, and right ATL would significantly decrease by aging during the recollection of memories associated with the SAG people as ingroup members, whereas age-dependent decreased activation in the right pSTS, hippocampus, and right ATL would be found in the recollection of memories associated with the DAG people as outgroup members. Second, functional connectivity between the CMS regions, including the dmPFC or precuneus, and the hippocampus-ATL regions would be significantly impaired by aging during the recollection of memories encoded with the SAG people as ingroup members, whereas age-dependent decreases in functional connectivity between the right pSTS and the hippocampus-ATL regions would be identified in the recollection of memories encoded with the DAG people as outgroup members.

## Materials and Methods

### Participants

Thirty-six healthy young adults (17 females; mean age: 22.42, SD: 1.66) and 36 healthy older adults (18 females; mean age: 66.22, SD: 3.01) participated in a 2-day experiment and were paid for their participation. All participants were right-handed, native Japanese speakers, with no history of neurological or psychiatric disease. All young participants were recruited from the Kyoto University community, and all older participants were recruited from Kyoto City Silver Human Resource Center. All participants gave informed consent to an experimental protocol approved by the Institutional Review Board (IRB) of the Graduate School of Human and Environmental Studies, the Kyoto University (29-H-8).

All participants performed several neuropsychological tests, including the FLANDERS handedness test (Nicholls et al., 2013; Okubo et al., 2014), the Japanese version of Montreal Cognitive Assessment (MoCA-J; Nasreddine et al., 2005; Fujiwara et al., 2010), and Center for Epidemiologic Studies Depression Scale (CES-D; Radloff, 1977; Shima, 1998). In addition, personality and social traits in each participant were assessed by NEO–FFI (Costa and MarcCrae, 1992; Shimonaka et al., 1999), University of California Los Angeles Loneliness Scale (UCLA-LS; Russell et al., 1978; Masuda et al., 2012), Subjective Well-Being Scale (SWBS; Sell and Nagpal, 1992; Ito et al., 2003), Interdependent Happiness Scale (IHS; Hitokoto and Uchida, 2015), Japanese version of Rosenberg Self-Esteem Scale (RSES-J; Rosenberg, 1965; Mimura and Griffiths, 2007), and Loyola Generativity Scale (LGS; Mcadams and Destaubin, 1992; Tabuchi et al., 2012). However, these personality and social trait scores were not included in all analyses of the present study.

One young participant and one older participant had MoCA-J scores lower than 2 SD below the mean scores in each group, and four young participants and five older participants had CES-D scores that extended far beyond the cutoff value. In addition, one young participant was found to have possible pathological changes (probable arachnoid cyst) in a structural MRI, one older participant did not complete her/his MRI scan due to a piece of internal metal, one older participant had difficulty communicating without hearing aids, one older participant had difficulty perceiving visual stimuli due to glaucoma, one young participant and one older participant showed head movement greater than 3 mm during the scanning, and one older participant showed more no-response trials than 2 SD above the mean number of no-response trials in all participants. Based on the exclusion criteria, behavioral and MRI data from 19 participants (seven young and 12 older adults, some of whom met several exclusion criteria) were excluded from all analyses. Thus, we analyzed data from 29 young adults (15 females; mean age: 22.45, SD: 1.72) and 24 older adults (11 females; mean age: 65.96, SD: 3.09) in the present study.

Neuropsychological test scores were compared between the two groups of 29 young and 24 older adults by two-sample *t*-tests (two-tailed). Significant differences between the two groups were identified in age [*t*_(__51__)_ = 64.78, *p* < 0.01, *d* = 17.88], education years [*t*_(__51__)_ = 5.87, *p* < 0.01, *d* = 1.62], and MoCA-J scores [*t*_(__51__)_ = 5.01, *p* < 0.01, *d* = 1.38]. However, we did not find a significant difference between the two groups in the FLANDERS handedness test [*t*_(__51__)_ = 0.37, *p* = 0.71, *d* = 0.10] and CES-D scores [*t*_(__51__)_ = 1.01, *p* = 0.32, *d* = 0.28]. Detailed profiles of young and older participants are summarized in Table 1.

**TABLE 1:** Participant characteristics.

	Young (SD)	Old (SD)	Group difference
**Age (years)**	22.45 (1.72)	65.96 (3.09)	Young < Old**
**Sex (male:female)**	14:15	13:11	
**Education**	16.14 (1.43)	13.33 (2.04)	Young > Old**
**MoCA-J**	28.41 (1.38)	26.04 (2.05)	Young > Old**
**FLANDERS**	9.83 (0.47)	9.88 (0.45)	n.s.
**CES-D**	8.69 (4.31)	7.58 (3.56)	n.s.

SD, standard deviation. FLANDERS, Japanese version of FLANDERS handedness questionnaire; MoCA-J, Japanese version of Montreal Cognitive Assessment; CES-D, Center for Epidemiologic Studies Depression Scale. **p < 0.01.

### Stimuli

A total of 120 Japanese words were selected from a database of two-letter Japanese Kanji words (Itsukushima et al., 1991). They were divided into three lists of 40 words, the two of which were used for target stimuli in two encoding conditions and the remaining one was used for distracter stimuli. These lists were counterbalanced across participants. In addition, each list was subdivided into two lists of 20 words, which were applied to two presenters in the encoding task described below. The attributions of imagery, concreteness, ease of learning, and frequency in each word were statistically equalized among these six lists. In one-way analyses of variance (ANOVA), no significant differences were found in attribution scores for imagery [*F*_(__5_,_119__)_ = 1.70, *p* = 0.14, η*^2^* = 0.07], concreteness [*F*_(__5_,_119__)_ = 0.91, *p* = 0.48, η*^2^* = 0.04], ease of learning [*F*_(__5_,_119__)_ = 0.59, *p* = 0.71, η*^2^* = 0.03], and frequency [*F*_(__5_,_119__)_ = 0.41, *p* = 0.84, η*^2^* = 0.02].

**FIGURE 1:**
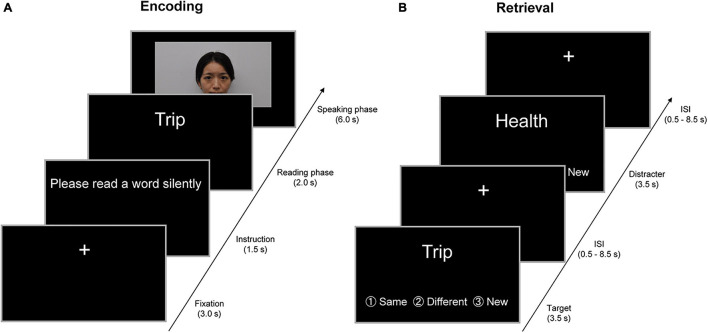
Example of the encoding and retrieval trials in a 2-day experiment. **(A)** Example of the encoding trials on the first day outside fMRI. In a trial of the encoding task, the instruction “Please read a word silently” was visually presented on a PC display for 1.5 s. In the subsequent “reading” phase, participants were visually presented with a target word for 2 s and were required to read the target word silently. In the “speaking” phase for 6 s, participants were randomly presented with a person belonging to the same age group (SAG) or different age group (DAG) in a movie clip. In this phase, participants initially watched the person in the movie and spoke the target word to the person. Subsequently, the person in the movie clip orally returned the same word to participants, and then participants spoke the same word again to the person in the movie clip. In the intertrial interval, a visual fixation was presented for 3 s at the center of the PC display. **(B)** Examples of the retrieval trials on the second day inside fMRI. In the retrieval task, participants were randomly presented with 80 learned and 40 new words one by one on an MRI-compatible PC display, and were required to judge whether each word had been learned from either SAG or DAG members or neither in the encoding task. Three response options (same generation, different generation, and new) were provided for the judgment. Each word was presented for 3.5 s, and then a visual fixation was shown during the interstimulus interval (ISI), which was jittered with variable durations (0.5–8.5 s). All verbal items were presented in Japanese. English is used here for illustration purposes only.

Target words were visually presented in a movie clip for approximately 18 min (see Figure 1) and were encoded through exchanges with four unfamiliar persons, two of whom were young adults and the other two of whom were older adults. To serve as a young adult in the movie clip, four graduate students (two females and two males) were recruited from the Kyoto University community, and four older adults (two females and two males) who were living outside the city of Kyoto appeared in the movie clip. To avoid sex effects on memory processes, target words were exchanged in the movie clip with persons of the same sex as the participants. The experimenter confirmed that the four persons in the movie clip were not familiar to the participants before the encoding task.

### Experimental Procedures

All young and older adults participated in a 2-day experiment in which participants were required to perform the encoding task outside the fMRI scanner on the first day, and then neural activation was measured in the retrieval task inside the scanner approximately 24 h after the encoding task (see Figure 1). No reference was made to a subsequent memory test during the encoding task, and hence, the encoding operation was incidental.

In the encoding task on the first day, the participants were visually presented with target words one by one by unfamiliar persons in the movie clip and were required to exchange each word with either a person who belonged to SAG or DAG. In a trial of the encoding task, the initial instruction “Please read a word silently” was visually presented on a PC display for 1.5 s. In the subsequent “reading” phase, the participants were visually presented with a target word for 2 s and were required to read the target word silently. In the “speaking” phase for 6 s, the participants were randomly presented with a SAG or DAG person in the movie clip. In this phase, the participants initially watched the person in the movie and spoke the target word to the person. Subsequently, the person in the movie clip orally returned the same word to the participants, and then participants spoke the same word again to the person in the movie clip. By these experimental operations, we created the virtual exchange of target words with the SAG and DAG people during the encoding task. In the intertrial interval (ITI), a visual fixation was presented for 3 s at the center of the PC display. All experimental procedures during the encoding task were recorded by a video camera.

In the retrieval task on the second day, which was performed in the MRI scanner approximately 24 h after the encoding task, the participants were randomly presented with 120 words one by one, including 80 target words and 40 new words, on an MRI-compatible PC display. The participants were required to judge whether each word was learned from the SAG or DAG person or not learned in the encoding task. Three response options (same generation, different generation, and new) were provided for the judgment, and the participants were instructed to show their response by pressing one of three buttons as soon as possible. Each word was presented for 3.5 s, and then a visual fixation was shown during the interstimulus interval (ISI), which was jittered with variable durations (0.5–8.5 s). The retrieval task was carried out in one run (approximately 12 min), and retrieval-related activation was measured by the event-related fMRI method. After the retrieval task, the participants evaluated their subjective feelings of attractiveness, trustworthiness, and familiarity of the two SAG and two DAG persons shown in the encoding task on a visual analog scale (VAS) that was 10 cm in length. The results of the VAS evaluations are summarized in the Supplementary Material.

### MRI Acquisition and Analysis

#### Data Acquisition

All MRI data were acquired by a MAGNETOM Verio 3.0 T MRI scanner (Siemens, Erlangen, Germany), which is located in Kokoro Research Center, Kyoto University. The stimulus presentation and recording of behavioral responses were controlled by MATLAB^®^ programs^1^ on a Windows PC. Experimental stimuli during the encoding task were presented on a Windows PC display, and experimental stimuli during the retrieval task were presented on an MRI-compatible display (Nordic Neuro Lab Inc., Bergen, Norway). During the retrieval task in the scanner, the participants viewed stimuli with a mirror attached to the head coil, and their responses during the retrieval task were recorded by three buttons in a four-button fiber-optic response device for the right hand (Current Designs Inc., Philadelphia, PA, United States). A set of earplugs helped reduce scanner noise, and foam pads were used to minimize head motions during scanning.

During the MRI scanning, three directional T1-weighted anatomical images were initially acquired to localize the subsequent functional and structural images. Second, 5-min resting-state functional images (TR = 2,000 ms, TE = 30 ms, flip angle = 87 degrees, FOV = 19.2 cm × 19.2 cm, matrix size = 64 × 64, slice thickness/gap = 3/1 mm, 33 horizontal slices collected by ascending order) and task-related functional images (TR = 2,000 ms, TE = 25 ms, flip angle = 75 degrees, FOV = 22.4 cm × 22.4 cm, matrix size = 64 × 64, slice thickness/gap = 3.5/0 mm, 39 horizontal slices collected by ascending order) were collected using a pulse sequence of gradient-echo echo-planar imaging (EPI), which is sensitive to blood oxygenation level-dependent (BOLD) contrasts. However, resting-state functional images were not included in the analysis of the present study. Finally, high-resolution T1-weighted structural images were obtained by MPRAGE (TR = 2,250 ms, TE = 3.51 ms, FOV = 25.6 cm × 25.6 cm, matrix size = 256 × 256, slice thickness/gap = 1.0/0 mm, 208 horizontal slices).

#### Preprocessing of fMRI Data

All MRI data were preprocessed and statistically analyzed by Statistical Parametric Mapping 12 (SPM12, Functional Imaging Laboratory, University College London, London, United Kingdom) implemented in MATLAB^®^ (see text footnote 1). Regarding the preprocessing, after discarding the initial four volumes, functional images in a retrieval run were initially corrected for slice timing, and then parameters of head motion were extracted from functional images. Second, high-resolution T1-weighted structural images for each participant were spatially aligned to functional images in the first scan of these functional images by the coregistration method. Third, structural images spatially aligned to functional images were spatially normalized into the tissue probability map (TPM) template in Montreal Neurological Institute (MNI) space, and parameters estimated by this spatial normalization were applied to all functional images (resampled resolution = 3.5 mm × 3.5 mm × 3.5 mm). Finally, normalized functional images were spatially smoothed by a Gaussian kernel of 8-mm FWHM.

#### Univariate Analysis of fMRI Data

Trials in which a target word was not spoken twice in the encoding task and those in which no response was shown in the retrieval task were excluded from all statistical analyses. Retrieval trials in which both target words and encoding conditions associated with the words were successfully recognized were categorized into Source Hit, and retrieval trials in which target words were successfully recognized but encoding conditions associated with the words were missed or in which target words were not recognized were regarded as Item-Only Hit and Miss. In addition, the Source Hit and Item-Only Hit + Miss trials were subdivided by encoding conditions, that is, trials encoded with the SAG people and those with the DAG people (Source Hit-SAG, Source Hit-DAG, Item-Only Hit + Miss-SAG, Item-Only Hit + Miss-DAG). Retrieval trials showing a response of “same generation” for distracter words were defined as False Alarm in the SAG response (False Alarm-SAG), retrieval trials showing a response of “different generation” for distracter words were categorized as False Alarm in the DAG response (False Alarm-DAG), and retrieval trials showing a “new” response for distracter words were categorized as Correct Rejection.

Statistical analyses of fMRI data were performed first at the individual level and then at the group level. In the individual-level (fixed-effect) analysis, trial-related activation during the retrieval task was modeled by convolving vectors of onset with a canonical hemodynamic response function (HRF) in the context of a general linear model (GLM). In this model, the timing when each word was presented was defined as the onset with an event duration of 0 s. Six parameters related to head motion were also included as confounding variables in this model. Trial-related activation in this model included six experimental conditions decided by encoding condition and retrieval performance (Source Hit-SAG, Source Hit-DAG, Item-Only Hit + Miss-SAG, Item-Only Hit + Miss-DAG, False Alarm-SAG + False Alarm-DAG, Correct Rejection) and one No-Response condition. Activation related to the Source Hit trial was identified by comparing trial-related activation of Source Hit with baseline activation in each condition of SAG and DAG, and the contrasts yielded a *t*-statistic in each voxel. These contrast images (Source Hit-SAG and Source Hit-DAG) were created for each participant.

In the group-level (random-effect) analysis, using two *t*-contrast images (Source Hit-SAG and Source Hit-DAG) obtained in the individual-level analyses, the Source Hit-related activation in each condition of SAG and DAG was analyzed by a two-way ANOVA with factors of the encoding condition (SAG and DAG) and subgroup (Young and Old). This ANOVA model was created by a flexible factorial design with a subject factor. Two types of statistical analyses were performed in this ANOVA. First, regions reflecting a significant aging effect on Source Hit-SAG vs. Source Hit-DAG were identified in an *F*-contrast of encoding condition-subgroup interaction masked inclusively by a *t*-contrast of [(Source Hit-SAG vs. Source Hit-DAG in Young) vs. (Source Hit-SAG vs. Source Hit-DAG in Old)] (*p* < 0.05). Second, to identify regions showing a significant aging effect on Source Hit-DAG vs. Source Hit-SAG, an *F*-contrast of encoding condition-subgroup interaction was masked inclusively by a *t*-contrast of [(Source Hit-DAG vs. Source Hit-SAG in Young) vs. (Source Hit-DAG vs. Source Hit-SAG in Old)] (*p* < 0.05).

In these analyses, the height threshold at the voxel level was corrected for multiple comparisons in the whole-brain and hypothesis-driven regions of interest (ROI) (FWE, *p* < 0.05). ROIs in the first analysis were set in the dmPFC, precuneus, hippocampus, and right ATL. In addition, the right pSTS, hippocampus, and right ATL ROIs were applied to the second analysis. ROIs in the hippocampus and precuneus were created bilaterally in the AAL ROI package (Tzourio-Mazoyer et al., 2002). The right ATL ROI covered the entire temporal pole (Brodmann’s area: BA 38), extending caudally up to the MNI coordinate *y* = −21 but did not include the superior temporal gyrus. In addition, this ROI included the fusiform and inferior temporal gyrus up to the MNI coordinate *y* = −25 and the fusiform gyrus alone up to the MNI coordinate *y* = −39 but did not include BA37 (Binney et al., 2010). The dmPFC ROI was extracted from the bilateral superior frontal and cingulate gyri in the AAL ROI package, which included the MNI coordinate *y* = 1 or more and *z* = 20 or more (Sugimoto and Tsukiura, 2018). The right pSTS ROI was defined as a sphere with a 5-mm radius at the center of a peak voxel in the pSTS (MNI coordinates: *x* = 62, *y* = −32, *z* = 0), which was identified in a previous study (Dodell-Feder et al., 2011).

#### Functional Connectivity Analysis of fMRI Data

Functional connectivity with the right hippocampus and right ATL regions, which showed significant activation in the univariate analyses, was investigated by a generalized form of context-dependent psychophysiological interaction (gPPI) analysis (McLaren et al., 2012). A one-run GLM, which included six experimental conditions, one No-Response condition, and confounding variables of six parameters related to head motion, was newly estimated in each participant, and seed regions of the right hippocampus and right ATL in this model were identified by volumes of interest (VOI) with a sphere of 5-mm radius centered on the peak voxel (right hippocampus: *x* = 29, *y* = −11, *z* = −25, and right ATL: *x* = 43, *y* = 7, *z* = −21). These VOIs as seed regions were anatomically masked by a sphere ROI of 10-mm radius centered on each peak voxel, and by each ROI of the right hippocampus extracted from the AAL ROI package (Tzourio-Mazoyer et al., 2002) and the right ATL mentioned above.

In the functional connectivity analysis, we employed the gPPI toolbox.^2^ This toolbox produces an individual-level (fixed-effect) model with three sets of columns, including: (1) condition-related regressors formed by convolving vectors of condition-related onsets with a canonical HRF; (2) time series of BOLD signals deconvolved from the seed region; and (3) PPI regressors as an interaction between (1) condition-related regressors and (2) time series of BOLD signals. Thus, the model in the present study included (1) condition-related regressors of the Source Hit-SAG, Source Hit-DAG, Item-Only Hit + Miss-SAG, Item-Only Hit + Miss-DAG, False Alarm-SAG + False Alarm-DAG, Correct Rejection, and No-Response condition; (2) BOLD signals of the right hippocampus or right ATL; and (3) PPI regressors of the Source Hit-SAG, Source Hit-DAG, Item-Only Hit + Miss-SAG, Item-Only Hit + Miss-DAG, False Alarm-SAG + Aalse Alarm-DAG, Correct Rejection, and No-Response condition. For each participant, the models for right hippocampal and right ATL seeds were estimated with confounding variables of six parameters related to head motion, and linear contrasts of PPI regressors were extracted as a *t*-contrast in each of Source Hit-SAG and Source Hit-DAG. Regions showing a significant effect in a *t*-contrast of PPI regressors reflected significant functional connectivity with each seed VOI. These *t*-contrasts of PPI regressors obtained in the individual-level analysis were applied to the group-level analysis. The right hippocampal VOI as a seed region was not significantly extracted from 6 young and 6 older adults. Thus, in the right hippocampal seed, PPI regressor contrasts obtained from 23 young and 18 older participants were analyzed in the group-level statistics. In the right ATL seed, significant VOI was not extracted from 3 young and 3 older adults; hence, PPI regressor contrasts identified in 26 young and 21 older adults were applied to the group-level analysis.

In the group-level (random-effect) analysis, *t*-contrast images reflecting significant functional connectivity with seed regions (right hippocampus and right ATL) in the Source Hit trials were compared between young and older adults by two-sample *t*-tests in each encoding condition (one-tailed). In this analysis, the height threshold at the voxel level was corrected for multiple comparisons in the whole-brain and each ROI of the dmPFC, precuneus, right pSTS, hippocampus, and right ATL regions (FWE, *p* < 0.05), which were explained above. Anatomical sites in all analyses were primarily defined using the SPM Anatomy toolbox (Eickhoff et al., 2005, 2006, 2007) and MRIcro.^3^

## Results

### Behavioral Results

Behavioral results are summarized in Table 2. Memory performance based on the Source Hit rate vs. False Alarm rate in the retrieval task was analyzed by a two-way mixed ANOVA with factors of subgroup (Young and Old) and encoding condition (SAG and DAG). ANOVA demonstrated a significant main effect of subgroup [*F*_(__1_,_51__)_ = 9.47, *p* < 0.01, η*_*p*_^2^* = 0.43] and a significant interaction between subgroup and encoding condition [*F*_(__1_,_51__)_ = 4.56, *p* < 0.05, η*_*p*_^2^* = 0.30]. However, a main effect of encoding condition was not significant [*F*_(__1_,_51__)_ = 0.22, *p* = 0.64, η*_*p*_^2^* = 0.07]. *Post hoc* tests by the Bonferroni method revealed a significant difference between Young and Old in the DAG condition (*p* < 0.05).

**TABLE 2:** Behavioral results.

	Young		Old	
	SAG (SD)	DAG (SD)	SAG (SD)	DAG (SD)
**Accuracy (proportion)**				
Source Hit	0.43 (0.12)	0.46 (0.10)	0.40 (0.08)	0.36 (0.11)
False Alarm	0.23 (0.10)	0.23 (0.10)	0.25 (0.11)	0.27 (0.12)
Source Hit vs. False Alarm	0.19 (0.13)	0.23 (0.12)	0.15 (0.14)	0.10 (0.13)
**Response time (ms)**				
Source Hit	2014.03 (429.18)	1999.74 (451.90)	2060.50 (349.39)	2118.34 (369.07)
False Alarm	2071.14 (441.15)	2151.46 (356.60)	2151.46 (356.60)	2188.76 (369.85)
**Number of trials**				
Source Hit	18.21 (4.11)	16.90 (4.93)	15.71 (3.29)	14.25 (4.20)
False Alarm	9.24 (3.94)	9.21 (3.74)	9.83 (4.51)	10.54 (4.89)

*SAG, same age group; DAG, different age group; SD, standard deviation.*

Response time (RT) data (ms) during the Source Hit trials in the retrieval task were analyzed by a two-way mixed ANOVA with factors of subgroup (Young and Old) and encoding condition (SAG and DAG). There were no significant main effects of subgroup [*F*_(__1_,_51__)_ = 0.58, *p* = 0.45, η*_*p*_^2^* = 0.10] and encoding condition [*F*_(__1_,_51__)_ = 0.54, *p* = 0.47, η*_*p*_^2^* = 0.10]. The interaction between these factors was also not significant [*F*_(__1_,_51__)_ = 1.47, *p* = 0.23, η*_*p*_^2^* = 0.17].

### fMRI Results

#### Univariate Analysis of fMRI Data

**FIGURE 2:**
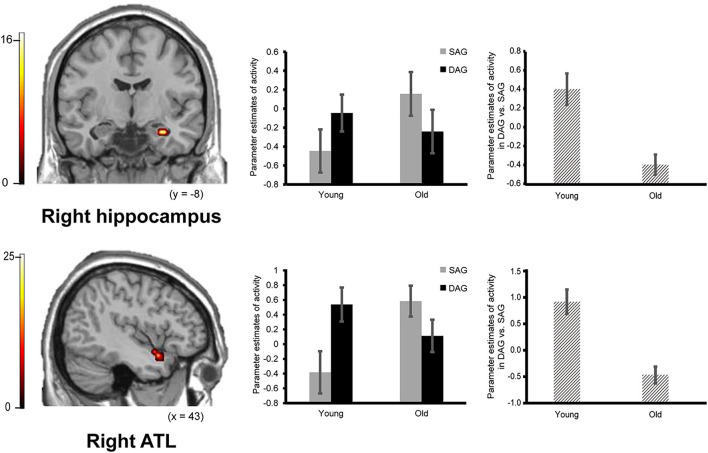
Activation in the right hippocampus (29, –11, –25, *p* = 0.026 corrected by FWE) and right ATL (anterior temporal lobe: 43, 7, –21, *p* = 0.003 corrected by FWE) reflecting a significant interaction between encoding condition and subgroup, in which an age-dependent decrease in activation was identified in a contrast of DAG (different age group) vs. SAG (same age group). Parameter estimates of activity in the bar graphs were extracted from peak voxels in each region. Error bars represent standard errors. The bar graphs in the middle of this figure showed parameter estimates of activity related to the Source Hit trials in each encoding condition, and the bar graphs in the right side showed differences in parameter estimates of activity related to the Source Hit trials between the DAG and SAG conditions. Results in this analysis were identified in fMRI data from 29 young adults and 24 older adults. In this analysis, the height threshold at the voxel level was corrected for multiple comparisons (FWE, *p* < 0.05) in each region of interest (ROI) of the bilateral hippocampi and right ATL which were defined anatomically.

Confirming our first prediction, the right hippocampus and right ATL showed age-dependent decreased activation during the successful retrieval of source memories encoded with the DAG people compared to those with the SAG people. However, age-dependent decreased activation in the successful retrieval of source memories related to the SAG people compared to the DAG people was not identified in any region (see Figure 2).

Successful retrieval activation of source memories was analyzed by a two-way ANOVA with subgroup (Young and Old) and encoding condition (SAG and DAG) as factors. In the first analysis, in which we examined regions showing age-dependent decreased activation in the successful retrieval of source memories encoded with the SAG people compared to the DAG people, an *F*-contrast reflecting a significant interaction between these factors was inclusively masked by a *t*-contrast of [(Source Hit-SAG vs. Source Hit-DAG in Young) vs. (Source Hit-SAG vs. Source Hit-DAG in Old)]. In this analysis, however, significant activation was not found in any regions in either whole-brain or ROI-based analyses. In the second analysis, which was conducted to find regions showing age-dependent decreased activation in the successful retrieval of source memories encoded with the DAG people compared to the SAG people, an *F*-contrast reflecting a significant interaction between these factors was inclusively masked by a *t*-contrast of [(Source Hit-DAG vs. Source Hit-SAG in Young) vs. (Source Hit-DAG vs. Source Hit-SAG in Old)]. This analysis of the predefined ROIs showed significant activation in the right hippocampus and right ATL (see Figure 2). In the whole-brain analysis, we did not find significant activation in any regions. Detailed results of the univariate analyses are summarized in Table 3.

**TABLE 3:** Regions reflecting significant interactions between factors of encoding condition and subgroup.

					MNI coordinates				
Regions			L/R	BA	*x*	*y*	*z*	*Z* score	k
**Age-related decrease in SAG vs. DAG**									
	**ROI-based analysis (dmPFC, precuneus, ATL, hippocampus)**								
		No significant activation was identified in any ROIs.							
	**Whole-brain analysis**								
		No significant activation was identified.							
**Age-related decrease in DAG vs. SAG**									
	**ROI-based analysis (pSTS, ATL, hippocampus)**								
		Hippocampus	R		29	−11	−25	3.61	3
		ATL	R	38	43	7	−21	4.36	10
	**Whole-brain analysis**								
		No significant activation was identified.							

*BA, Brodmann area; k, cluster size; L, left; R, right; ROI, region of interest; ATL, anterior temporal lobe; dmPFC, dorsomedial prefrontal cortex; pSTS, posterior superior temporal sulcus.*

#### Functional Connectivity Analysis of fMRI Data

**FIGURE 3:**
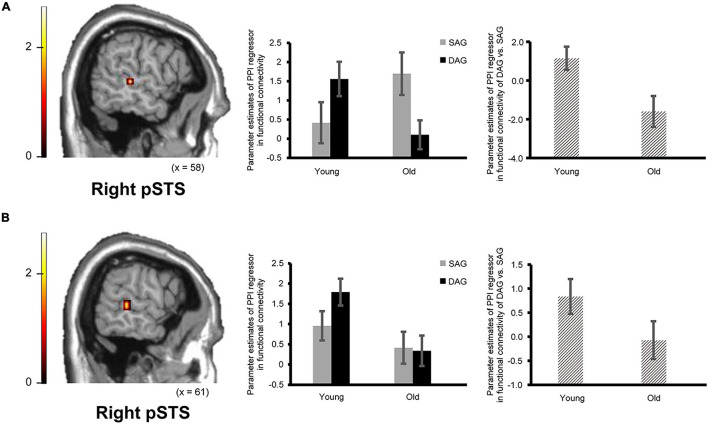
Regions showing an age-dependent decrease in functional connectivity during the successful retrieval of source memories in the DAG conditions. **(A)** The right pSTS (posterior superior temporal sulcus: 61, –32, 0, *p* = 0.045 corrected by FWE) showing an age-dependent decrease in functional connectivity with the right hippocampal seed. Results in this analysis were identified in fMRI data from 23 young adults and 18 older adults, from whom a seed volume of interest (VOI) was extracted. **(B)** The right pSTS (64, –35, 4, *p* = 0.024 corrected by FWE) showing an age-dependent decrease in functional connectivity with the right ATL (anterior temporal lobe) seed. Results in this analysis were identified in fMRI data from 26 young adults and 21 older adults, from whom a seed VOI was extracted. Parameter estimates of PPI regressors in the bar graphs were extracted from peak voxels in each region of the pSTS showing significant functional connectivity. Error bars represent standard errors. The bar graphs in the middle of this figure showed parameter estimates of PPI regressors related to the Source Hit trials in each encoding condition, and the bar graphs in the right side showed differences in parameter estimates of PPI regressors related to the Source Hit trials between the DAG and SAG conditions. In these analyses, the height threshold at the voxel level was corrected for multiple comparisons (FWE, *p* < 0.05) in each ROI of the right pSTS, which was defined anatomically.

Confirming our second prediction, functional connectivity of the right hippocampus or the right ATL with the right pSTS during the successful retrieval of source memories encoded with the DAG people significantly decreased in the Old subgroup compared to the Young subgroup (see Figure 3). However, functional connectivity between the hippocampus/ATL and the CMS regions during the successful retrieval of source memories encoded with the SAG people was not different between the Young and Old subgroups.

In the functional connectivity analysis with the right hippocampal and ATL seeds, in which significant activation was identified in the univariate analysis, age-dependent decreases in functional connectivity in the Source Hit trials were investigated by two-sample *t*-tests between the Young and Old subgroups in each condition (one-tailed). In the DAG condition, an age-dependent decrease in functional connectivity with the right hippocampal seed was found in the right middle temporal gyrus (pSTS) in the ROI-based analysis. However, the whole-brain analysis in this condition did not show any regions reflecting significant functional connectivity with the right hippocampal seed. The functional connectivity analysis with the right ATL seed in the DAG condition showed a significant aging effect on the right middle temporal gyrus (pSTS) in the ROI-based analysis and on the left calcarine region in the whole-brain analysis. In the SAG condition, we did not find any regions showing significant age-dependent decrease in functional connectivity with the right hippocampal seed or the right ATL seed in either whole-brain or ROI-based analyses. Detailed results of the functional connectivity analyses are summarized in Table 4.

**TABLE 4:** Regions showing age-related decrease in functional connectivity in each encoding condition.

					MNI coordinates				
Regions			L/R	BA	*x*	*y*	*z*	*Z* score	k
**SAG condition**									
**Right hippocampus seed**									
	**ROI-based analysis (dmPFC, precuneus, ATL)**								
		No significant activation was identified in any ROIs.							
	**Whole-brain analysis**								
		No significant activation was identified.							
**Right ATL seed**									
	**ROI-based analysis (dmPFC, precuneus, hippocampus)**								
		No significant activation was identified in any ROIs.							
	**Whole-brain analysis**								
		No significant activation was identified.							
**DAG condition**									
**Right hippocampus seed**									
	**ROI-based analysis (pSTS, ATL)**								
		Middle temporal gyrus	R		61	−32	0	2.35	1
	**Whole-brain analysis**								
		No significant activation was identified.							
**Right ATL seed**									
		**ROI-based analysis (pSTS, hippocampus)**							
	Middle temporal gyrus		R		64	−35	4	2.61	2
	**Whole-brain analysis**								
		Calcarine cortex	L		−24	−53	11	5.05	2

*BA, Brodmann area; k, cluster size; L, left; R, right; ROI, region of interest; ATL, anterior temporal lobe; dmPFC, dorsomedial prefrontal cortex; pSTS, posterior superior temporal sulcus.*

## Discussion

Two major findings emerged from the present study. First, the right hippocampus and right ATL showed an age-dependent decrease in activation during the successful retrieval of source memories encoded with the DAG people as outgroup members compared to the SAG people as ingroup members. In the successful retrieval of source memories encoded with the SAG people as ingroup members compared to the DAG people as outgroup members, however, an age-dependent decrease in activation was not identified in any region. Second, an age-dependent decrease in functional connectivity of the right hippocampus or the right ATL with the right pSTS was significant during the successful retrieval of source memories encoded with the DAG people as outgroup members, whereas a significant aging effect on functional connectivity with the right hippocampus or the right ATL was not found in any region during the successful retrieval of source memories encoded with the SAG people as ingroup members. These findings suggest that functional networks including the hippocampus related to the recollection process of episodic memories, the ATL related to the processing of social knowledge, and the pSTS related to the processing of other people could be impaired by aging in the recollection of memories associated with the DAG people as outgroup members. These findings are discussed in separate sections below.

### Age-Dependent Decreased Activation During the Recollection of Memories Associated With Outgroup Members Categorized by Age-Group Membership

The first main finding of the present study was that activation in the right hippocampus and right ATL significantly decreased by aging during the successful recollection of source memories encoded with the DAG people as outgroup members compared to the SAG people as ingroup members. This finding suggests that the hippocampus and ATL, which contribute to the retrieval of associations between face and person-related social knowledge, are more involved in the recollection of memories associated with the DAG people as outgroup members than the recollection of memories associated with the SAG people as ingroup members and that the functionality of these regions is impaired by aging.

In the present study, we found that the right hippocampus and right ATL showed an age-dependent decrease in activation during the retrieval of source memories encoded with the DAG people as outgroup members compared to the SAG people as ingroup members. This finding is consistent with previous studies, which have demonstrated that recollection-related hippocampal activation significantly decreases with age (Shing et al., 2010; Koen and Yonelinas, 2014). For example, one fMRI study reported that activation in the hippocampus related to recollection significantly decreased in older adults compared to young adults, whereas activation related to familiarity in the entorhinal and perirhinal cortices was relatively preserved in older adults (Daselaar et al., 2006). In another fMRI study, an age-dependent decrease in hippocampal activation was identified in the recollection of autobiographical memories (St. Jacques et al., 2012). In addition, ATL activation in the present study is consistent with a previous fMRI study that reported an age-dependent decline in correlations between hippocampal and ATL activation during the retrieval of associations between face and face-related social knowledge, including people’s name and job title (Tsukiura et al., 2011). There is also functional neuroimaging evidence that ATL activation reflects the successful retrieval of person-related semantics as a type of social knowledge (Wang et al., 2017). The importance of the ATL in the processing of social knowledge has been consistently identified in functional neuroimaging studies and neuropsychological studies with brain-damaged patients (for review, see Olson et al., 2007; Olson et al., 2013). In the present study, the right hippocampus and right ATL showed significantly greater activation during the successful recollection of memories associated with the DAG people as outgroup members than that of memories associated with the SAG people as ingroup members, and activation in these regions significantly decreased by aging. Given our behavioral data that older adults showed worse retrieval performance than young adults only in the DAG condition, the hippocampus and ATL could play an important role in forming associations between face and social knowledge in the recollection of memories encoded with an outgroup member, and activation in these regions could be impaired by aging.

### Age-Related Differences in Functional Connectivity During the Recollection of Memories Associated With Outgroup Members Categorized by Age-Group Membership

The second main finding of the present study was that an age-dependent decrease in functional connectivity was found between the hippocampus/ATL and the pSTS during the successful retrieval of source memories in the DAG condition. The finding suggests that functional networks including the hippocampus related to the retrieval of source memories, the pSTS related to social cognition of others, and the ATL related to the processing of social knowledge contribute to the recollection of memories associated with the DAG people as outgroup members, and that this mechanism related to the recollection of memories associated with outgroup members could decrease as a result of aging.

In the present study, functional connectivity of the right hippocampus or the right ATL with the right pSTS was significantly lower in older adults than in young adults during the successful retrieval of source memories encoded with the DAG people as outgroup members. This finding might be explained by evidence from previous studies showing that social cognition networks, including the pSTS, anterior cingulate cortex, ATL and temporal-parietal junction (TPJ), contribute to the mentalizing of others’ mental states (for review, see Gallagher and Frith, 2003; Do and Telzer, 2019). For example, one fMRI study demonstrated that the ATL region, which showed greater activation during face processing than during scene or object processing, was functionally connected with the pSTS in resting-state fMRI scanning (Simmons et al., 2010). In another study, activation in the pSTS was modulated by a change in facial identity or expression (Fox et al., 2009). In addition, there is cognitive neuroscience evidence that the ATL is involved in the processing of person-related social knowledge (for review, see Olson et al., 2013), and that an age-dependent decrease in interacting mechanisms between the ATL and hippocampus are identified in the retrieval of social knowledge (name or job title) associated with faces (Tsukiura et al., 2011). Thus, functional connectivity between the ATL and pSTS in the DAG condition of the present study could reflect the mentalizing of others’ mental states by referring to social knowledge about the DAG people, and an age-dependent decrease in functional networks including the hippocampus, ATL and pSTS suggests that the recollection of memory for the DAG people by the mentalizing of others’ mental states with reference to social knowledge could be impaired in older adults.

### No Aging Effect on Activation and Functional Connectivity During the Recollection of Memories Associated With Ingroup Members Categorized by Age-Group Membership

Inconsistent with our prediction, an age-dependent decrease in activation and functional connectivity was not identified in the successful recollection of source memories encoded with the SAG people as ingroup members compared to the DAG people as outgroup members. This finding implies that the intergroup bias in memory is relatively preserved in older adults.

There is psychological evidence in which intergroup bias, such as the own-age bias, is commonly observed between young and older adults in several measures, including ingroup favoritism, perceived similarity, social distance, outgroup homogeneity, and self-stereotyping (Chasteen, 2005). The preserved own-age bias in older adults was also found in episodic memory research in which both young and older adults showed a significant own-age bias in name recall with better memory for names associated with faces of their own age, as compared to other-aged faces (Strickland-Hughes et al., 2020). In addition, one fMRI study reported that the amygdala showed significant activation during perceiving faces of the SAG people compared to the DAG people in both young and older groups (Wright et al., 2008). Behavioral data in the present study revealed that the Source Hit responses in the DAG condition were significantly lower in older adults than in young adults, whereas the Source Hit responses in the SAG condition were not different between young and older adults. Thus, the present finding that an age-dependent decrease in activation and functional connectivity during the successful recollection of memories in the SAG condition compared to the DAG condition was not identified in any regions could reflect the preserved intergroup bias related to the own-age bias during the recollection of source memories in older adults.

## Conclusion

In the present study, using event-related fMRI, we investigated age-related differences in neural mechanisms underlying the successful recollection of memories encoded with the SAG people as ingroup members and with the DAG people as outgroup members. The results demonstrated that activation in the right hippocampus and right ATL significantly decreased with age during the successful source retrieval of memories encoded with the DAG people as outgroup members compared to the SAG people as ingroup members. However, no region showed age-dependent decreased activation during the successful source retrieval of memories associated with the SAG people as ingroup members compared to the DAG people as outgroup members. In addition, an age-dependent decrease in functional connectivity with the hippocampus or the ATL was significantly identified in the pSTS in the DAG condition, whereas functional connectivity with the hippocampus or the ATL in the SAG condition showed no significant aging effect on any regions. These findings suggest that functional networks including the hippocampus, ATL, and pSTS, which contribute to the recollection of memories for the DAG people by the mentalizing of others’ mental states with reference to social knowledge, could be impaired in older adults compared to young adults, whereas neural mechanisms underlying the intergroup bias during the recollection of source memories for the SAG people could be relatively preserved in older adults.

## Data Availability Statement

The raw data supporting the conclusions of this article will be made available by the authors, without undue reservation.

## Ethics Statement

The studies involving human participants were reviewed and approved by Institutional Review Board (IRB) for Human Information Research of the Graduate School of Human and Environmental Studies, Kyoto University. The patients/participants provided their written informed consent to participate in this study.

## Author Contributions

ET and TT designed the study and analyzed the data. ET conducted the fMRI experiment and wrote the original draft of the manuscript. TT reviewed and edited the manuscript and obtained funding for the experiment. Both authors contributed to the article and approved the submitted version.

## Conflict of Interest

The authors declare that the research was conducted in the absence of any commercial or financial relationships that could be construed as a potential conflict of interest.

## Publisher’s Note

All claims expressed in this article are solely those of the authors and do not necessarily represent those of their affiliated organizations, or those of the publisher, the editors and the reviewers. Any product that may be evaluated in this article, or claim that may be made by its manufacturer, is not guaranteed or endorsed by the publisher.

## Footnotes

1
www.mathworks.com

2
www.nitrc.org/projects/gppi

3
https://people.cas.sc.edu/rorden/mricro/mricro.html
