# Supplementary material for: Effects of Aging on the Neural Mechanisms Underlying the Recollection of Memories Encoded by Social Interactions With Persons in the Same and Different Age Groups
Source: Front Behav Neurosci. 2021 Sep 10;15:743064. doi: 10.3389/fnbeh.2021.743064 (PMC8462460; doi:10.3389/fnbeh.2021.743064)
Supplement: Supplementary file 1 [file Table_1.DOCX]

SUPPLEMENTARY MATERIAL

| **SUPPLEMENTARY TABLE\|** Results of the VAS evaluation (cm) for the SAG and DAG persons in movie clips during encoding | | | | | | |
| --- | --- | --- | --- | --- | --- | --- |
|  | **Attractiveness** | | **Trustworthiness** | | **Familiarity** | |
|  | **Young (SD)** | **Old (SD)** | **Young (SD)** | **Old (SD)** | **Young (SD)** | **Old (SD)** |
| **Male participants** | | | | | | |
| **Young A** | 5.31 (0.98) | 4.02 (1.67) | 5.66 (1.73) | 4.96 (1.79) | 6.24 (1.23) | 5.11 (1.73) |
| **Young B** | 3.80 (1.39) | 3.75 (1.32) | 3.81 (1.69) | 3.98 (1.23) | 3.86 (2.41) | 3.94 (2.16) |
| **Old A** | 4.48 (2.25) | 4.10 (1.71) | 4.85 (2.38) | 5.25 (1.39) | 4.81 (2.48) | 5.40 (1.72) |
| **Old B** | 3.90 (2.18) | 4.41 (1.27) | 4.22 (2.40) | 5.13 (1.77) | 4.21 (2.50) | 5.38 (1.87) |
| **Female participants** | | | | | | |
| **Young A** | 6.83 (2.03) | 6.47 (2.04) | 6.32 (2.43) | 6.81 (1.33) | 6.28 (2.50) | 7.01 (1.91) |
| **Young B** | 6.10 (1.23) | 5.11 (1.43) | 5.50 (2.15) | 5.91 (1.49) | 6.14 (2.12) | 4.70 (1.85) |
| **Old A** | 5.59 (1.70) | 5.61 (1.52) | 6.27 (1.79) | 6.43 (1.49) | 6.15 (2.09) | 6.66 (1.74) |
| **Old B** | 3.73 (1.79) | 3.78 (1.37) | 4.38 (2.23) | 5.32 (1.51) | 3.03 (2.60) | 4.43 (2.23) |
| SD, standard deviation; VAS, visual analogue scale. | | | | | | |
